# SERPINB1 overexpression protects myocardial damage induced by acute myocardial infarction through AMPK/mTOR pathway

**DOI:** 10.1186/s12872-022-02454-7

**Published:** 2022-03-15

**Authors:** Hongliang Wang, Jun Hua, Shiyuan Chen, Ying Chen

**Affiliations:** 1 Department of Cardiovasology, First People’s Hospital of Jinan, Jinan, 250000 Shandong People’s Republic of China; 2 Department of Clinical Laboratory, Gaotang County People’s Hospital, Liaocheng, 252800 Shandong People’s Republic of China; 3 Department of Breast and Thyroid Surgery, Dongying People’s Hospital, Dongying, 257091 Shandong People’s Republic of China; 4 Department of Clinical Laboratory, Central Hospital of Shengli Oilfield, No. 31 Jinan Road, Dongying, 257000 Shandong People’s Republic of China

**Keywords:** SERPINB1, Acute myocardial infarction, AMPK/mTOR pathway, Apoptosis

## Abstract

**Background:**

SERPINB1 is involved in the development of a variety of diseases. The purpose of this study was to explore the effect of SERPINB1 on acute myocardial infarction (AMI).

**Methods:**

Serum SERPINB1 level of AMI patients was measured for receiver operating characteristic curve analysis. The AMI rat model was constructed to observe myocardial damage, and the H9C2 cell oxygen glucose deprivation (OGD) model was constructed to detect cell viability. Transthoracic echocardiography was used to assess the cardiac function. TTC staining and HE staining were used to detect pathologic changes of myocardial tissues. The apoptosis of myocardial tissues and cells were measured by TUNLE staining and flow cytometry assay. CCK-8 assay to measure cell viability. SERPINB1 expression was measured by qRT-PCR. Protein expression was measured by western blot.

**Results:**

The serum SERPINB1 level was down-regulated in AMI patients. AMI modeling reduced the SERPINB1 expression level, induced inflammatory cells infiltrated, and myocardial apoptosis. OGD treatment inhibited cell viability and promoted apoptosis. The AMPK/mTOR pathway was inhibited in AMI rats and OGD-treated H9C2 cells. Overexpression of SERPINB1 reduced infarct size and myocardial apoptosis of AMI rats, inhibited apoptosis of H9C2 cells, and activated AMPK/mTOR pathway. However, AMPK inhibitor Dorsomorphin reversed the protective effect of SERPINB1 on myocardial cells.

**Conclusion:**

SERPINB1 overexpression relieved myocardial damage induced by AMI via AMPK/mTOR pathway.

## Background

Acute myocardial infarction (AMI) is myocardial necrosis caused by acute and persistent ischemia and hypoxia in coronary arteries [1]. The main feature of AMI is apoptosis of myocardial cells [2, 3]. AMI is one of the most serious ischemic heart diseases. The morbidity and mortality of AMI remains high although the mortality rate of AMI has been declining over the past decades [4]. Therefore, the therapeutic measures involved in the mechanism of myocardial cell apoptosis will provide a new therapeutic method for the treatment of AMI.

Serine protease inhibitors (serpins) are a protein superfamily with conserved tertiary structure [5, 6]. Due to the lack of secretory signal sequence, SERPINB1 belongs to the B serpins, which mainly resides in the cytoplasm of neutrophils and monocytes [7]. El Ouaamari et al. have shown that SERPINB1 promotes pancreatic β cell proliferation in multiple species as a circulating factor secreted by the liver [8]. Study of Yao et al. has found that SERPINB1 can alleviate acute lung injury in liver transplantation [9]. Inhibition of SERPINB1 attenuated the protective effects of FoxO1 on diabetic nephropathy in vitro [10]. Besides, serpins may prevent acute cardiovascular syndromes by inhibiting serine proteases [11]. Also, SerpinA1 was shown to directly affect apoptosis by inhibition of caspase-1 and caspase-3 [12, 13]. Alpha-1-antitrypsin (the protein encoded by SerpinA1) treatment reduced infarct sizes in AMI mice [13]. Furthermore, SerpinB9 was demonstrated to inhibit cell apoptosis and protect against atherosclerotic lesion progression [14, 15]. However, the molecular mechanism of SERPINB1 acting on AMI still needs further detailed study.

Myocardial apoptosis induced by AMI may be related to the activation or inhibition of signaling pathways. Na et al. has demonstrated that gambogic acid protects against myocardial damage in MI rats by activating NF-κB and p38 pathways [16]. Liu et al. have found that inhibition of AMPK aggravated cell injury in OGD-mediated cardiomyocytes [17]. Additionally, Hua et al. have found that AMPK/mTOR signaling pathway is involved in AMI process [18]. A study of Wang et al. reported that activation of AMPK attenuated isoprenaline-induced myocardial fibrosis in vivo and decreased collagen deposition [19]. Furthermore, activation of the AMPK/mTOR pathway attenuates myocardial injury and cardiac insufficiency following MI [20]. However, whether SERPINB1 could alleviate myocardial apoptosis induced by AMI by regulating the AMPK/mTOR pathway has not been fully explored.

In this study, we measured serum SERPINB1 levels in AMI patients and healthy volunteers. Then we established the AMI rat model and cell model by LAD and OGD treatment to detect the effect of SERPINB1 on AMI rats and OGD-treated myocardial cells. The results showed that SERPINB1 in AMI patients was down-regulated. Overexpression of SERPINB1 could significantly alleviate myocardial damage and apoptosis induced by AMI through activating the AMPK/mTOR pathway. The results of our study might provide new ideas for the therapy of AMI.

## Methods

### Patients and samples

In this prospective study, we analyzed 40 cases AMI patients admitted to the Department of Cardiovasology, First people’s Hospital of Jinan and 40 matched healthy volunteers between August, 2017 and November, 2019. All patients received no therapy prior to blood collection, and healthy volunteers had no medical history of cardiovascular diseases. The diagnosis of AMI is based on the Third Universal Definition of Myocardial Infarction [21]. The inclusion criteria were as follows: Under these conditions any one of the following criteria meets the diagnosis for MI: Detection of a rise and/or fall of cardiac biomarker values [preferably cardiac troponin (cTn)] with at least one value above the 99th percentile upper reference limit (URL) and with at least one of the following: Symptoms of ischaemia; New or presumed new significant ST-segmenteT wave (STeT) changes or new left bundle branch block (LBBB); Development of pathological Q waves in the ECG; Imaging evidence of new loss of viable myocardium or new regional wall motion abnormality; Identification of an intracoronary thrombus by angiography. Venous blood was collected directly from patients with AMI and healthy volunteers upon admission, and serum samples were obtained by centrifugation. SERPINB1 level of serum samples were measured for receiver operating characteristic (ROC) curve analysis. All the experiments were approved by the Ethics Committee of the hospital, and informed consents were received from all participants.

### The creation and treatment of AMI rat model

Adult male SD rats (200–220 g) were obtained from Medical Experimental Animal Center of Guangdong Province. The rat model of AMI was established by the left anterior descending (LAD) coronary artery. The rats were anesthetized by intraperitoneal injection of 50 mg/kg pentobarbital sodium and underwent thoracotomy to expose cardiac tissue. Left main coronary artery was ligated with a 9–0 prolene suture at 1 mm below the ostium. Sham rats underwent the same surgical procedure without ligating LAD coronary artery.

In vivo transfection experiment, AMI rats were transfected with SERPINB1 overexpressed plasmid pcDNA3.1 (Thermo Fisher Scientific, Waltham, MA, USA) SERPINB1 (AMI + pc- SERPINB1) or its negative control (AMI + pc-NC).

### Cell culture and oxygen–glucose deprivation (OGD) treatment

Rat myocardial cell H9C2 was purchased from mibio (Shanghai, China). To simulate AMI, the H9C2 cells were cultured in non-serum and non-glucose medium at 37 °C for 0, 6, 12 or 24 h under 94% N_2_/5% CO_2_/1% O_2_.

In vitro transfection experiment, H9C2 cells were transfected with pc-SERPINB1 or pc-NC. 48 h after transfection, the cells were exposed to OGD model. In vitro validation experiment, H9C2 cells were treated with pc-SERPINB1 and AMPK inhibitor Dorsomorphin (Dors). 48 h after treatment, the cells were exposed to OGD model.

### Transthoracic echocardiography (TTE)

24 h after AMI modeling, rats were anesthetized by pentobarbital sodium (40 mg/kg) intraperitoneally. The TTE was used to assess the cardiac function of rats. The ultrasonic echocardiographic system was used to assess the rats' cardiac function after anesthetization. The left ventricular enddiastolic diameter (LVEDD) and left ventricular endsystolic diameter (LVESD) were measured from three consecutive cardiac cycles. The ejection fraction (EF) and fractional shortening (FS) were calculated by the following equation: EF = (LVEDV-LVESV)/LVESV × 100%; FS = (LVEDD-LVESD)/LVESD × 100%. Then, rats were sacrificed by cervical dislocation to collect myocardial tissue for follow-up experiments.

### Hematoxylin–eosin (H&E) staining

The pathological changes of myocardial tissues were detected by H&E staining. The rats were deeply anesthetized with an overdose of pentobarbital (100 mg/kg) intraperitoneally and then sacrificed via dislocation. The hearts of rats were harvested and rinsed with ice-cold PBS. The myocardial tissue was fixed with 4% paraformaldehyde and embedded in paraffin. Then the myocardial tissue was cut into 4 μm sections. The sectioning was performed with H&E to evaluate the pathological changes of myocardial tissue. The stained sections were visualized using a light microscope at × 200 magnification.

### TUNEL staining

The TUNEL staining was used to evaluate apoptosis of myocardial tissues. Myocardial tissue was immobilized with 4% paraformaldehyde and embedded in paraffin. Then tissues were sectioned into 4 μm slices. After dewaxing and rehydration, TUNEL kit (Shanghai Ruisai Biotechnology Co., Ltd., Shanghai, China) was used strictly followed the method of the kit. Finally, hematoxylin was used for restaining, and the results were observed in five randomly selected visual fields under a light microscope at × 200 magnification.

### Flow cytometry assay

The cell apoptosis was detected by the FITC-Annexin V Apoptosis Detection Kit (Sigma Aldrich, St. Louis, MO, USA).H9C2 cells were washed with cold PBS and resuspended with 500 µL binding buffer. Then the cells were stained with 5 µL Annexin V-FITC and 10 µL PI for 15 min in the dark. The cell apoptosis was measured on a flow cytometer (BD, Franklin Lakes, NJ, USA).

### qRT-PCR

The expression level of SERPINB1 in myocardial tissues or cells was detected by qRT-PCR. TRIZOL regent was used to extract the total RNA of myocardial tissues and cells (Invitrogen, Carlsbad, CA, USA). The sequences were as follows: SERPINB1, 5′-CGGCCTGTCGGTTTTCAC-3′ (forward) and 5′-TCTCACTCAACGCCAGGAAC-3′ (reverse); GAPDH, 5′-GAAGGTCGGAGTCAACGGATT-3′ (forward) and 5′-TTCCCGTTCTCAGCCATGT-3′ (reverse). SuperScriptTM IV first-strand Synthesi System (Invitrogen, Carlsbad, CA, USA) was used to synthesize cDNA. The expression of SERPINB1 was detected by using the SYBR Green kit (Invitrogen, USA). PCR amplification was implemented as follows: 95 °C for 5 min, 40 cycles of 95 °C for 5 s, and 61 °C for 30 s. GAPDH was used as a reference gene for SERPINB1 expression calculation. RNA expression was calculated by the 2^−ΔΔCt^ method.

### Western blot

To obtain the total proteins, myocardial tissues or cells were lysed with RIPA lysate containing protease inhibitors (Thermo Fisher Scientific, Waltham, MA, USA). The protein was subjected to SDS-PAGE, and then was transferred onto PVDF membrane. The membranes were blocked by nonfat milk and then incubated at the temperature of 4 °C overnight with primary antibodies (Bax, 1:1000, 14,796; Bcl-2, 1:1000, 4228 s, Cell Signaling Technology, Danvers, MA, USA; SERPINB1, 1:1000, sc-293462; caspase-3, 1:1000, sc-271759, Santa Cruz Biotechnology, Santa Cruz, CA, USA; AMPK, 1:1000, SAB4502329, p-AMPK, 1:1000, SAB4503754; mTOR, 1:1000, T2949, p-mTOR, 1:1000, SAB4504476, Sigma Aldrich, St. Louis, MO, USA). Subsequently, membranes were incubated with secondary antibodies (Sigma Aldrich, St. Louis, MO, USA) properly under room temperature. The western blots were visualized with ECL detection reagents (GE Healthcare, Little Chalfont, Buckinghamshire, UK).

#### Statistical analysis

Data were shown as means ± SD. Statistical analysis was performed with SPSS 23.0 (SPSS, Chicago, USA) and GraphPad Prism 7.0 (GraphPad Prism Software, Inc., San Diego, US). One-way analysis of variance test was used for comparison among groups, and unpaired Student's t-test was used for comparison between groups. The receiver operating characteristic (ROC) curve was used to analyze the clinical diagnostic value of the detection of serum SERPINB1. A P-value of < 0.05 was considered statistically significant.

## Results

### The expression of SERPINB1 was down-regulated in AMI patients

**Fig. 1:**
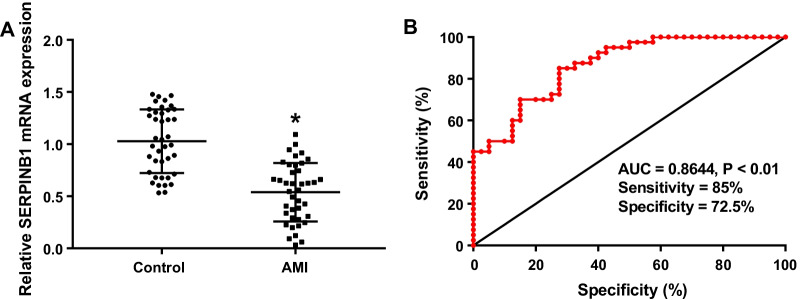
The expression of LncRNA SERPINB1 was down-regulated in acute myocardial infarction (AMI) patients. **A** The expression of SERPINB1 in AMI patients (n = 40) and health controls (n = 40) was detected by qRT-PCR. **B** Receiver operating characteristic (ROC) curve for AMI patients based on SERPINB1. *P < 0.05

We measured the expression of serum SERPINB1 in 40 AMI patients as well as 40 health controls by qRT-PCR. The data suggested that SERPINB1 expression in serum of AMI patients was evidently lower than that of control (Fig. 1A, *P* < 0.05). Besides, we evaluated the diagnostic efficacy of SERPINB1 by ROC curve. The result of ROC curve indicated that the area under the curve (AUC) was 0.8644 (sensitivity = 85%, specificity = 72.5%, *P* < 0.01). Serum SERPINB1 might be a biomarker to predict AMI in a way.

### Overexpression of SERPINB1 alleviated the impairment of myocardial function caused by AMI

**Fig. 2:**
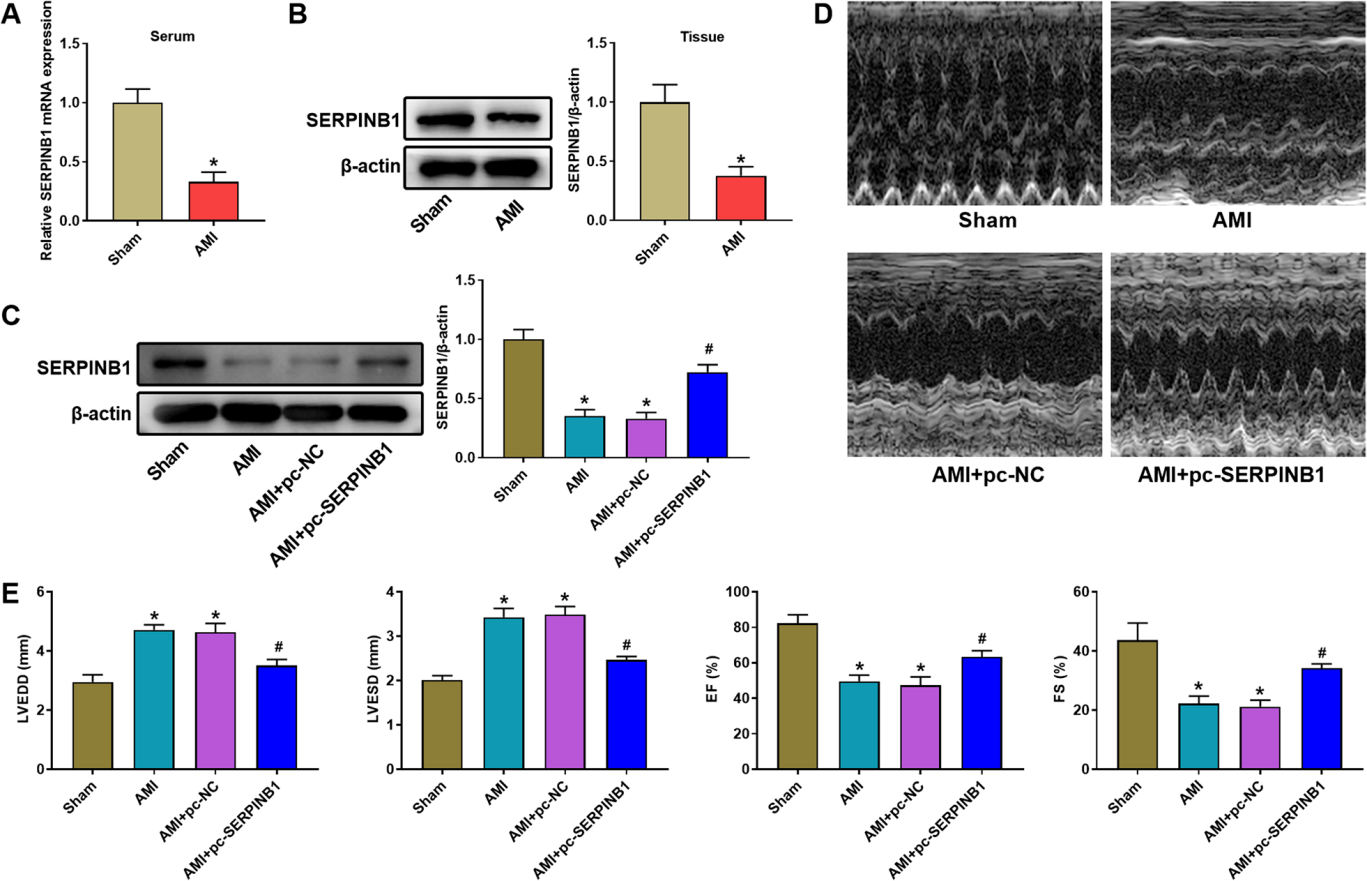
Overexpression of SERPINB1 alleviates the impairment of myocardial function caused by acute myocardial infarction (AMI). **A** The relative expression of SERPINB1 in serum of rats was measured by qRT-PCR. **B** The relative expression of SERPINB1 in myocardial tissues of rats was measured by western blot. **C** The relative expression of SERPINB1 after pcDNA3.1 SERPINB1 transfection was measured by western blot. **D** The representative images of echocardiographic assessment of hearts after pcDNA3.1 SERPINB1 transfection were measured by transthoracic echocardiography (TTE). (E) The analysis results of echocardiographic assessment of hearts after left anterior descending (LAD) ligation. *P < 0.05 vs. Sham group. ^#^*P* < 0.05 vs. AMI + pc-NC group

We measured the expression of SERPINB1 after the establishment the AMI rat model by LAD. The data of qRT-PCR and western blot indicated that AMI modeling markedly decreased the expression of SERPINB1 both in serum and myocardial tissues of rats (Fig. 2A, B, *P* < 0.05). To explore the role of SERPINB1 on AMI, the SERPINB1 overexpressed plasmid pcDNA3.1 SERPINB1 (pc-SERPINB1) was transfected into AMI rats. The data suggested that the SERPINB1 in myocardial tissues was significantly enhanced in AMI + pc-SERPINB1 group in comparison with AMI + pc-NC group (Fig. 2C, *P* < 0.05). The analysis results of TTE suggested that the myocardial function of rats in AMI group was remarkably destroyed compared with Sham group through the analysis of LVEDD, LVESD, FS, and EF (Fig. 2D, E, *P* < 0.05). The LVEDD and LVESD were remarkably increased by AMI modeling, while the FS and EF were significantly decreased (Fig. 2D, E, *P* < 0.05). Distinctly, overexpression of SERPINB1 reduced LVEDD and LVESD and enhanced FS and EF (Fig. 2D, E, *P* < 0.05).

### Overexpression of SERPINB1 inhibited AMPK/mTOR pathway and relieved myocardial injury induced by AMI modeling

**Fig. 3:**
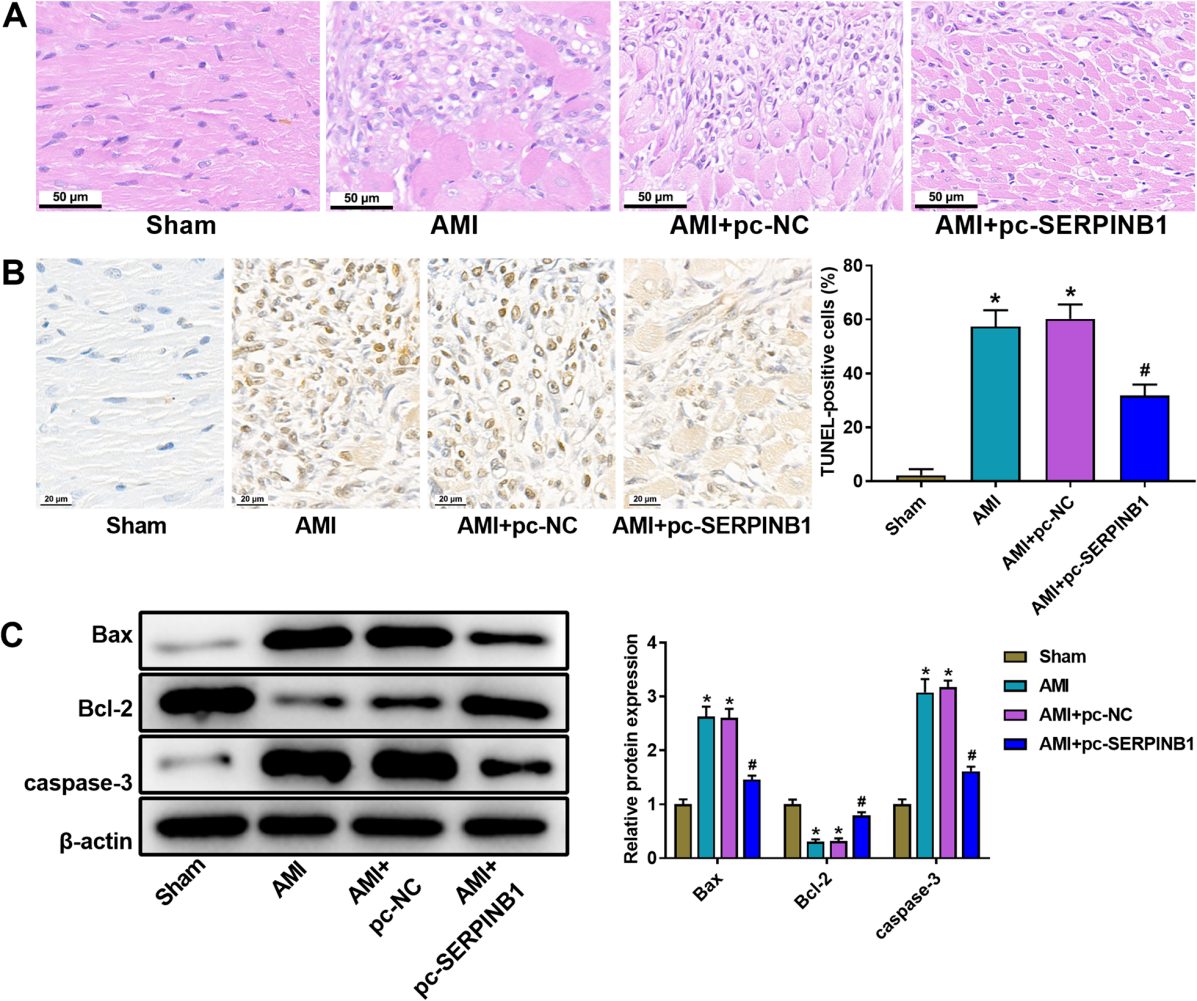
Overexpression of SERPINB1 relieves myocardial injury induced by acute myocardial infarction (AMI) modeling. **A** The representative images of HE staining assessment of myocardial tissues after pcDNA3.1 SERPINB1 transfection (× 200). **B** The representative images and analysis results of TUNEL staining assessment of myocardial tissues after pcDNA3.1 SERPINB1 transfection (Scale bar = 25 μm). **C** The protein expression of Bax, Bcl-2, and caspase-3 in myocardial tissues after pcDNA3.1 SERPINB1 transfection was measured by western blot. *P < 0.05 vs. Sham group. ^#^P < 0.05 vs. AMI + pc-NC group

The H&E staining was used to detect the pathological changes of myocardial tissue after transfection with pc-SERPINB1. The results indicated that AMI modeling caused visibly myocardial injury, and the myocardial injury of AMI rats was markedly remitted by pc-SERPINB1 (Fig. 3A). We detected the apoptosis of myocardial tissues by TUNEL staining. The TUNEL data suggested that the TUNEL-positive cells was markedly increased in AMI group in comparison of Sham group (Fig. 3B, *P* < 0.05). Overexpression of SERPINB1 could markedly reduce the number of TUNRL-positive cells compared with AMI + pc-NC group (Fig. 3B, *P* < 0.05). Besides, we measured the protein expression of Bax, Bcl-2, and caspase-3 by western blot. The results suggested that the protein expression of Bax and caspase-3 was remarkably higher and the Bcl-2 protein expression was lower in AMI group than that in Sham group (Fig. 3C, *P* < 0.05). SERPINB1 overexpression significantly reduced the protein expression of Bax and caspase-3, while enhanced Bcl-2 protein expression (Fig. 3C, *P* < 0.05).

### OGD inhibited SERPINB1 expression and induced apoptosis of myocardial cells

**Fig. 4:**
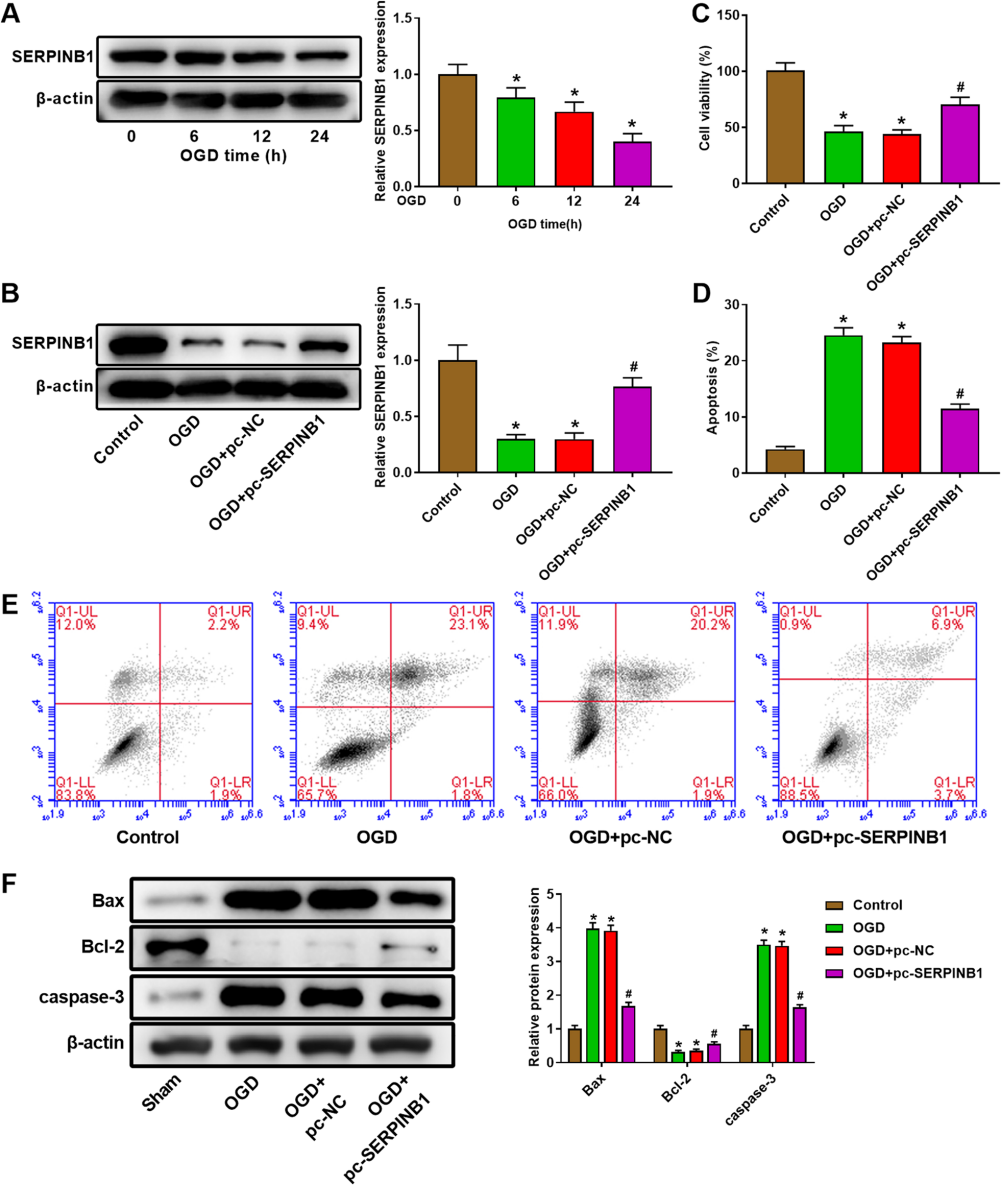
Overexpression of SERPINB1 reduced apoptosis of myocardial cells induced by oxygen–glucose deprivation (OGD) treatment. **A** The expression of SERPINB1 of myocardial cells H9C2 after OGD treatment for 0, 6, 12 or 24 h was measured by western blot. *P < 0.5 vs. 0 h. **B** The expression of SERPINB1 of myocardial cells H9C2 after pcDNA3.1 SERPINB1 transfection was measured by western blot. **C** The cell viability of myocardial cells H9C2 after pcDNA3.1 SERPINB1 transfection was measured by CCK-8 assay. **D**, **E** The cell apoptosis of myocardial cells H9C2 after pcDNA3.1 SERPINB1 transfection was measured by flow cytometry. **F** The protein of Bax, Bcl-2, and caspase-3 of myocardial cells H9C2 after pcDNA3.1 SERPINB1 transfection was measured by western blot. *P < 0.05 vs. Control group. ^#^P < 0.05 vs. OGD + pc-NC group

The OGD cell model was constructed to inquire the influence of SERPINB1 on myocardial cells. The expression of SERPINB1 in H9C2 cells was detected by western blot after OGD treatment at 0, 6, 12 or 24 h. The data showed that OGD treatment reduced SERPINB1 expression and the inhibition of OGD on SERPINB1 expression was enhanced with the extension of treatment time (Fig. 4A, *P* < 0.05). As shown in Fig. 4B, the expression of SERPINB1 was significantly enhanced by pc-SERPINB1 (*P* < 0.05). The CCK-8 assay was used to measured cell viability of H9C2 after pc-SERPINB1 transfection. The result suggested that OGD treatment suppressed cell viability of H9C2 cells, and the inhibiting effect of OGD on H9C2 cells could be remitted by SERPINB1 overexpression (Fig. 4C, *P* < 0.05). In addition, the cell apoptosis of H9C2 cells was detected by flow cytometry analysis. We found that the apoptosis of H9C2 cells was markedly increased by OGD treatment (Fig. 4D, E, *P* < 0.05). Overexpression of SERPINB1 inhibited cell apoptosis compared with OGD + pc-NC group (Fig. 4D, E, *P* < 0.05). The western blot result suggested that OGD treatment remarkably reduced protein expression of Bax and caspase-3 and enhanced Bcl-2 protein expression (Fig. 4F, *P* < 0.05). The protein expression of Bax and caspase-3 was markedly lower and the protein expression of Bcl-2 was significantly higher in OGD + pc-SERPINB1 group than that in OGD + pc-NC group (Fig. 4F, *P* < 0.05).

### Overexpression of SERPINB1 activated AMPK/mTOR pathway

**Fig. 5:**
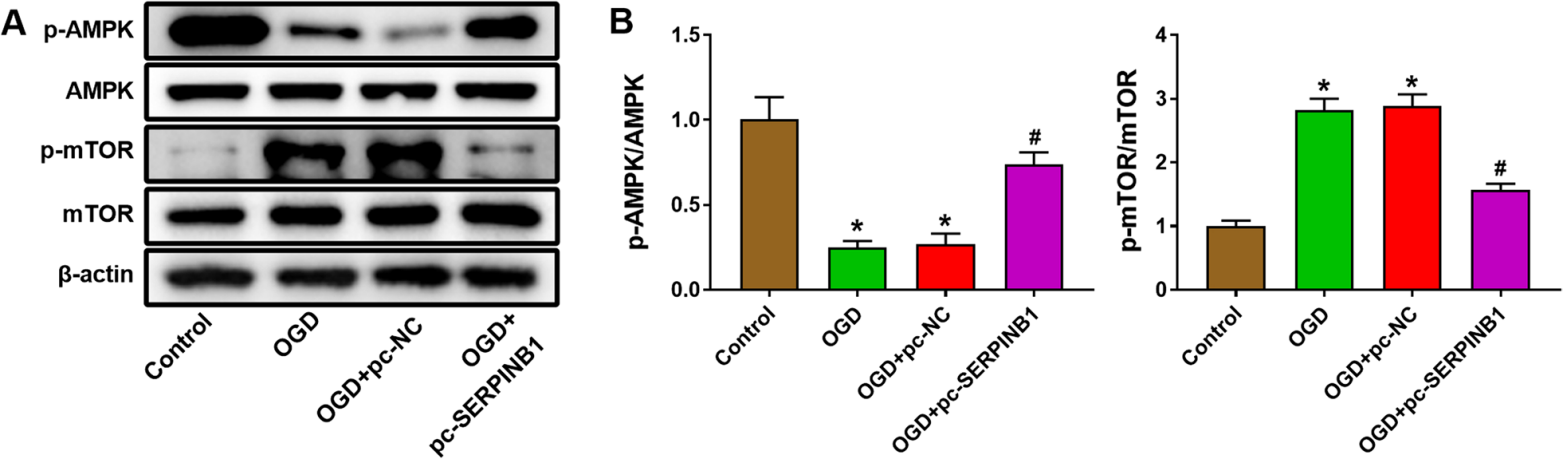
Overexpression of SERPINB1 activated AMPK/mTOR pathway. **A** The protein and phosphorylation levels of AMPK and mTOR of myocardial cells H9C2 after pcDNA3.1 SERPINB1 transfection was measured by western blot. **B** The analysis results of protein and phosphorylation levels of AMPK and mTOR. *P < 0.05 vs. Control group. ^#^*P* < 0.05 vs. OGD + pc-NC group

In this study, we found abnormal protein expression of p-AMPK/AMPK and p-mTOR/mTOR in myocardial cells H9C2 after OGD treatment. The AMPK/mTOR pathway was inhibited by OGD treatment. The western blot data indicated that OGD treatment markedly reduced the protein expression of p-AMPK/AMPK and enhanced the protein expression of p-mTOR/mTOR (Fig. 5, *P* < 0.05). To ascertain the effect of SERPINB1 on the AMPK/mTOR pathway, we measured the expression of p-AMPK/AMPK and p-mTOR/mTOR after pc-SERPINB1 transfection. The results indicated that overexpression of SERPINB1 activated the AMPK/mTOR pathway. The protein expression of p-AMPK/AMPK was increased and the protein expression of p-mTOR/mTOR was decreased in OGD + pc-SERPINB1 group compared with OGD + pc-NC group (Fig. 5, *P* < 0.05).

### Inhibition of the AMPK/mTOR pathway eliminated the protective effect of SERPINB1 on H9C2 cells

**Fig. 6:**
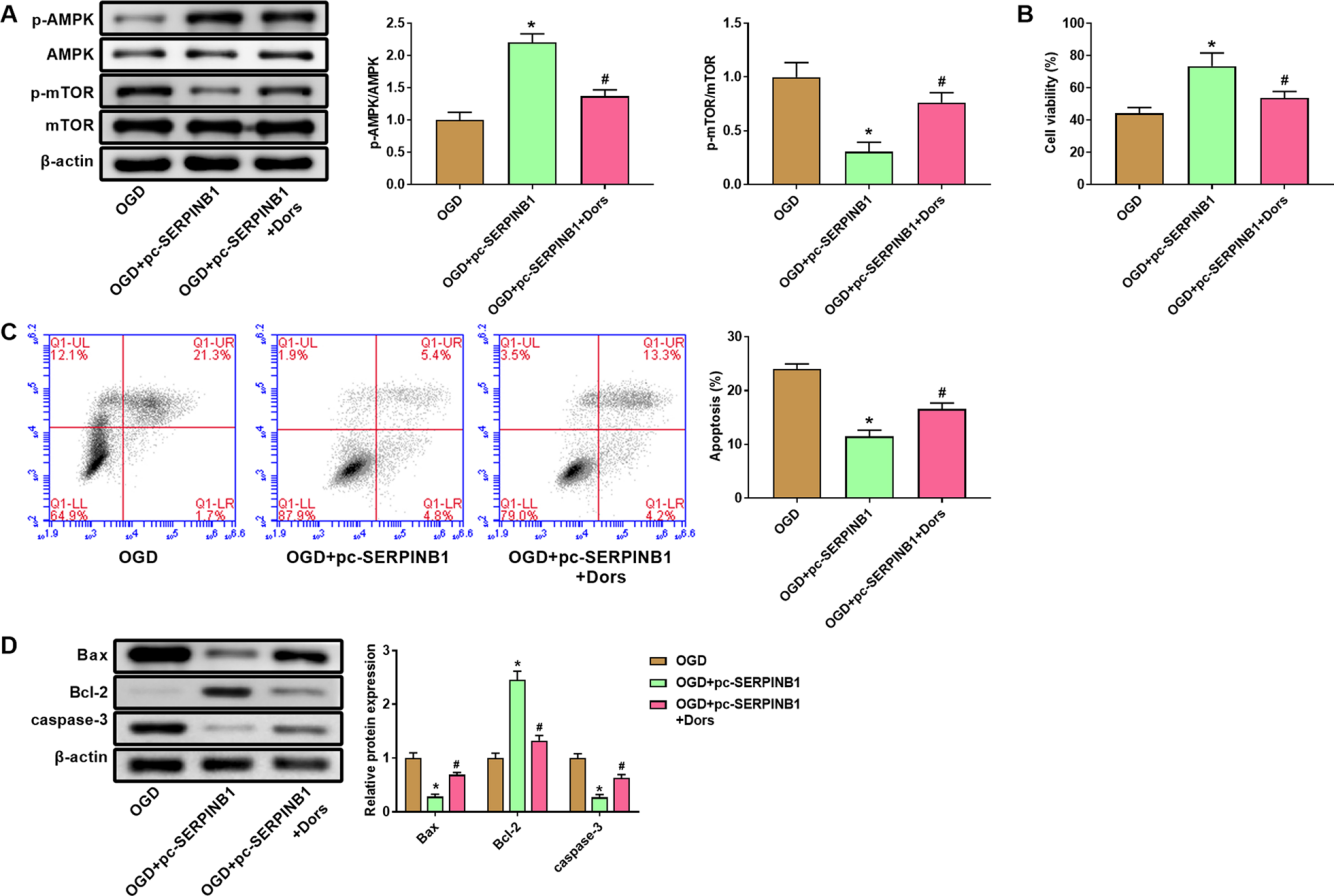
Inhibition of AMPK/mTOR pathway aggravated cell apoptosis induced by oxygen–glucose deprivation (OGD) of H9C2 cells. **A** The protein and phosphorylation levels of AMPK and mTOR of myocardial cells H9C2 after Dors treatment was measured by western blot. **B** The cell viability of myocardial cells H9C2 after Dors treatment was measured by CCK-8 assay. **C** The cell apoptosis of myocardial cells H9C2 after Dors treatment was measured by flow cytometry. **D** The protein of Bax, Bcl-2, and caspase-3 of myocardial cells H9C2 after Dors treatment was measured by western blot. *P < 0.05 vs. OGD group. ^#^*P* < 0.05 vs. OGD + pc-SERPINB1 group

In order to verify the role of AMPK/mTOR pathway on OGD-treated myocardial cells, we cultured H9C2 cells with the AMPK inhibitor Dors before OGD treatment. The data in Fig. 6A suggested that AMPK inhibitor Dors significantly inhibited the protein expression of p-AMPK/AMPK while enhanced p-mTOR/mTOR expression (*P* < 0.05). CCK-8 result showed that Dors treatment reduced cell viability of H9C2 dells (Fig. 6B, *P* < 0.05). Besides, we measured apoptosis of H9C2 cells by flow cytometry. The data suggested that cell apoptosis of OGD + pc-SERPINB1 + Dors group was remarkably increased compared with OGD + pc-SERPINB1 group (Fig. 6C, *P* < 0.05). In compaison with OGD + pc-SERPINB1 group, the protein expression of Bax and caspase-3 in OGD + pc-SERPINB1 + Dors group was markedly increased, while Bcl-2 protein was decreased (Fig. 6D, *P* < 0.05).

## Discussion

AMI is one of the most serious cardiovascular diseases with high mortality despite improvements in treatment strategies over the past decade [4]. In our study, we found that the SERPINB1 expression level was remarkably down-regulated in AMI patients, AMI rats as well as OGD-treated myocardial cells. AMI modeling resulted in obviously myocardial injury in myocardial tissue. The apoptosis rates of H9C2 cells increased by OGD treatment. Besides, the AMPK/mTOR pathway was inhibited in OGD-treated myocardial cells.

Abnormal SERPINB1 expression affects the progression of many diseases. Benarafa et al. have demonstrated that serpinB1 is vital for the maintaining the health of bone marrow pool in acute lung injury, knockdown of SERPINB1 increased the apoptosis and necrosis of purified bone marrow neutrophil [22]. SERPINB1 can promote the proliferation of porcine pancreatic stem cells (pPSCs) and play an important role in the transformation of pPSCs into insulin secreting cells [23]. Additionally, SERPINB1 gene knockdown aggravated lung injury of mice with orthotopic autologous liver transplantation (OALT), and administration of recombinant SERPINB1 protein attenuated cell apoptosis in the lung after OALT [9]. Moreover, Alpha-1-antitrypsin treatment in AMI mice increased LVEDD and LVESD, and reduced LVEF [24]. In this study, we found that serum SERPINB1 was significantly down-regulated in AMI patients. The result of ROC analysis indicated that the level of serum SERPINB1 might be a biomarker for the diagnosis of AMI. We constructed AMI rat model and cell model by LAD method and OGD treatment, respectively. The expression levels of SERPINB1 in AMI rat serum and OGD-treated H9C2 cells were both markedly suppressed. These data indicated that SERPINB1 was involved in the procession of AMI. Overexpression of SERPINB1 significantly reduced the myocardial injury caused by AMI modeling. The number of TUNEL-positive cells in myocardial tissue of AMI rats was significantly reduced after overexpression of SERPINB1. Besides, overexpression of SERPINB1 markedly enhanced cell viability and reduced the apoptosis of H9C2 cells treated by OGD. The above results suggested that high expression of SERPINB1 could reduce myocardial apoptosis and alleviate myocardial damage that caused by AMI.

The AMPK/mTOR pathway is widely taken part in the development of numerous diseases, including ischemic acute kidney injury, diabetes, and cardiac dysfunction [25–27]. Study of Hu et al. has indicated that gAPN prevented cell apoptosis of chondrocytes induced by H2O2 through activation of AMPK/mTOR pathway [28]. Chen et al. have proved that AMPK activator AICAR strengthens the anti-apoptotic effect of Berberine on Müller cells stimulated with high glucose [29]. Overexpression of SIRT3 exhibited a protective role in rotenone-induced Parkinson's disease cell model by activating AMPK/mTOR pathway [30]. A study of Zhou et al. demonstrated that exedin-4 inhibits cardiac hypertrophy by activating the AMPK/mTOR signaling pathway [31]. Apocynum leaf extract can marked increase the p-AMPK but decrease the mTOR protein expression to reduce blood lipid levels in rats with atherosclerosis and delay atherosclerotic progression [32]. Xu et al. have suggested that leonurine inhibited myocardial apo ptosis and improved myocardial function in rat model by activating the PI3K/AKT/GSK3β pathway [33]. Yi et al. has found that inhibition of RhoA/ROCK pathway significantly mitigated AMI-induced myocardial apoptosis in mice [34].The analogical results were obtained in our study. We found that AMI modeling inhibited the AMPK/mTOR pathway and induced myocardial apoptosis. The p-AMPK level was remarkably reduced while p-mTOR level was markedly enhanced. SERPINB1 overexpression enhanced p-AMPK level, and increased p-AMPK level significantly reduced myocardial injury and myocardial apoptosis of AMI rats. The cell viability of OGD-treated H9C2 cells was significantly strengthened and the cell apoptosis was suppressed by activating p-AMPK. Besides, the AMPK inhibitor Dors accelerated cell apoptosis of H9C2 cells treated by OGD. These data suggested that activation of AMPK/mTOR pathway might improve myocardial damage by inhibiting myocardial apoptosis.

## Conclusion

Overexpression of SERPINB1 alleviated myocardial damage induced by AMI in rats and apoptosis of OGD-treated H9C2 cells through activating AMPK/mTOR pathway. Our findings indicated that SERPINB1 might be a hopeful therapeutic target for AMI. However, we currently only explored the protective effect of SERPINB1 on AMI rats and cardiomyocytes. The role of SERPINB1 in human cardiomyocytes still needs further research. Additionally, extensive in vivo and in vitro studies are required before the results can be applied to clinical studies.

## Data Availability

The datasets used and analyzed during the current study are available from the corresponding author on reasonable request. (E-mail: yingchencycy@163.com).

## Abbreviations

AMI: Acute myocardial infarction
serpins: Serine protease inhibitors
OGD: Oxygen glucose deprivation
ROC: Receiver operating characteristic
TTE: Transthoracic echocardiography
LAD: Left anterior descending
AUC: Area under the curve
LVEDD: Left ventricular enddiastolic diameter
LVESD: Left ventricular endsystolic diameter
EF: Ejection fraction
FS: Fractional shortening
H&E: Hematoxylin–eosin
pPSCs: Porcine pancreatic stem cells
OALT: Orthotopic autologous liver transplantation

## References

[1] Erdal C, Karakülah G, Fermancı E, Kunter I, Silistreli E, Canda T (2012). Early biventricular molecular responses to an acute myocardial infarction. Int J Med Sci.

[2] Konstantinidis K, Whelan R, Kitsis R (2012). Mechanisms of cell death in heart disease. Arterioscler Thromb Vasc Biol.

[3] Marchant D, Boyd J, Lin D, Granville D, Garmaroudi F, McManus B (2012). Inflammation in myocardial diseases. Circ Res.

[4] Reed G, Rossi J, Cannon C (2016). Acute myocardial infarction. Lancet.

[5] Silverman GA, Bird PI, Carrell RW, Church FC, Coughlin PB, Gettins PGW (2001). The serpins are an expanding superfamily of structurally similar but functionally diverse proteins. J Biol Chem.

[6] Richardson J, Viswanathan K, Lucas A (2006). Serpins, the vasculature, and viral therapeutics. Front Biosci.

[7] Remold-O'Donnell E, Chin J, Alberts M (1992). Sequence and molecular characterization of human monocyte/neutrophil elastase inhibitor. Proc Natl Acad Sci USA.

[8] El Ouaamari A, Dirice E, Gedeon N, Hu J, Zhou JY, Shirakawa J (2016). SerpinB1 promotes pancreatic β cell proliferation. Cell Metab.

[9] Yao W, Li H, Luo G, Li X, Chen C, Yuan D (2017). SERPINB1 ameliorates acute lung injury in liver transplantation through ERK1/2-mediated STAT3-dependent HO-1 induction. Free Radical Biol Med.

[10] Liang X, Su Y, Huo Y (2020). Forkhead box protein O1 (FoxO1)/SERPINB1 ameliorates ROS production in diabetic nephropathy. Food Sci Nutr.

[11] Johan K, Paul HAQ, Ilze B (2013). Anti-apoptotic serpins as therapeutics in cardiovascular diseases. Cardiovasc Hematol Disord Drug Targets.

[12] Petrache I, Fijalkowska I, Medler TR, Skirball J, Cruz P, Zhen L (2006). alpha-1 antitrypsin inhibits caspase-3 activity, preventing lung endothelial cell apoptosis. Am J Pathol.

[13] Toldo S, Seropian I, Mezzaroma E, Van Tassell B, Salloum F, Lewis E (2011). Alpha-1 antitrypsin inhibits caspase-1 and protects from acute myocardial ischemia–reperfusion injury. J Mol Cell Cardiol.

[14] Kummer JA, Micheau O, Schneider P, Bovenschen N, Broekhuizen R, Quadir R (2007). Ectopic expression of the serine protease inhibitor PI9 modulates death receptor-mediated apoptosis. Cell Death Differ.

[15] Hendel A, Cooper D, Abraham T, Zhao H, Allard MF, Granville DJ (2012). Proteinase inhibitor 9 is reduced in human atherosclerotic lesion development. Cardiovasc Pathol.

[16] Na D, Aijie H, Bo L, Zhilin M, Long Y (2017). Gambogic acid exerts cardioprotective effects in a rat model of acute myocardial infarction through inhibition of inflammation, iNOS and NF-κB/p38 pathway. Exp Therapeutic Med.

[17] Liu C-Y, Zhou Y, Chen T, Lei J-C, Jiang X-J (2021). AMPK/SIRT1 pathway is involved in arctigenin-mediated protective effects against myocardial ischemia-reperfusion injury. Front Pharmacol.

[18] Hua J, Liu Z, Liu Z, An D, Lai W, Zhan Q (2018). Metformin increases cardiac rupture after myocardial infarction via the AMPK-MTOR/PGC-1α signaling pathway in rats with acute myocardial infarction. Med Sci Monit.

[19] Wang L, Yuan D, Zheng J, Wu X, Wang J, Liu X (2019). Chikusetsu saponin IVa attenuates isoprenaline-induced myocardial fibrosis in mice through activation autophagy mediated by AMPK/mTOR/ULK1 signaling. Phytomedicine.

[20] Yan J, Yan J-Y, Wang Y-X, Ling Y-N, Song X-D, Wang S-Y (2019). Spermidine-enhanced autophagic flux improves cardiac dysfunction following myocardial infarction by targeting the AMPK/mTOR signalling pathway. Br J Pharmacol.

[21] Thygesen K, Alpert JS, Jaffe AS, Simoons ML, Chaitman BR, White HD (2012). Third universal definition of myocardial infarction. Glob Heart.

[22] Benarafa C, LeCuyer TE, Baumann M, Stolley JM, Cremona TP, Remold-O'Donnell E (2011). SerpinB1 protects the mature neutrophil reserve in the bone marrow. J Leukoc Biol.

[23] Xu S, Qin D, Yang H, He C, Liu W, Tian N (2020). SerpinB1 promotes the proliferation of porcine pancreatic stem cells through the STAT3 signaling pathway. J Steroid Biochem Mol Biol.

[24] Toldo S, Seropian IM, Mezzaroma E, Van Tassell BW, Salloum FN, Lewis EC (2011). Alpha-1 antitrypsin inhibits caspase-1 and protects from acute myocardial ischemia–reperfusion injury. J Mol Cell Cardiol.

[25] Jeon S-M (2016). Regulation and function of AMPK in physiology and diseases. Exp Mol Med.

[26] Tang M, Lusco M, Abate M, Levine J. Preconditioning mice with activators of AMPK ameliorates ischemic acute kidney injury in vivo. Am J Physiol Renal Physiol. 2016;311:ajprenal.00541.02015. 10.1152/ajprenal.00541.2015 27252492

[27] Zhang J, Zhao P, Quan N, Wang L, Chen X, Cates C (2017). The endotoxemia cardiac dysfunction is attenuated by AMPK/mTOR signaling pathway regulating autophagy. Biochem Biophys Res Commun.

[28] Hu J, Cui W, Ding W, Gu Y, Wang Z, Fan W (2017). Globular adiponectin attenuated H2O2-induced apoptosis in rat chondrocytes by inducing autophagy through the AMPK/ mTOR pathway. Cell Physiol Biochem Int J Exp Cell Physiol Biochem Pharmacol.

[29] Chen H, Ji Y, Yan X, Su G, Chen L, Xiao J (2018). Berberine attenuates apoptosis in rat retinal Müller cells stimulated with high glucose via enhancing autophagy and the AMPK/mTOR signaling. Biomed Pharmacother.

[30] Zhang M, Deng Y-N, Zhang J-Y, Liu J, Li Y-B, Su H (2018). SIRT3 protects rotenone-induced injury in SH-SY5Y cells by promoting autophagy through the LKB1-AMPK-mTOR pathway. Aging Dis.

[31] Zhou Y, He X, Chen Y, Huang Y, Wu L, He J (2015). Exendin-4 attenuates cardiac hypertrophy via AMPK/mTOR signaling pathway activation. Biochem Biophys Res Commun.

[32] Lü L, Zhang D, Sun B, Hu Y, Yan M, Liu K (2017). Apocynum leaf extract inhibits the progress of atherosclerosis in rats via the AMPK/mTOR pathway. Die Pharmazie Int J Pharmaceut Sci.

[33] Xu L, Jiang X, Wei F, Zhu H (2018). Leonurine protects cardiac function following acute myocardial infarction through anti-apoptosis by the PI3K/AKT/GSK3β signaling pathway. Mol Med Rep.

[34] Yi Z, Ke J, Wang Y, Cai K (2020). Fluvastatin protects myocardial cells in mice with acute myocardial infarction through inhibiting RhoA/ROCK pathway. Exp Ther Med.

